# MicroRNAs and intellectual disability (ID) in Down syndrome, X-linked ID, and Fragile X syndrome

**DOI:** 10.3389/fncel.2013.00041

**Published:** 2013-04-15

**Authors:** Wei-Hong Siew, Kai-Leng Tan, Maryam Abbaspour Babaei, Pike-See Cheah, King-Hwa Ling

**Affiliations:** ^1^NeuroBiology and Genetics Group, Genetic Medicine Research Centre, Faculty of Medicine and Health Sciences, Universiti Putra MalaysiaUPM Serdang, Malaysia; ^2^Clinical Genetics Unit, Department of Obstetrics and Gynaecology, Faculty of Medicine and Health Sciences, Universiti Putra MalaysiaUPM Serdang, Malaysia; ^3^Department of Human Anatomy, Faculty of Medicine and Health Sciences, Universiti Putra MalaysiaUPM Serdang, Malaysia

**Keywords:** Down syndrome, brain development, cognitive function, Fragile X syndrome, X-linked genetic disease, non-coding RNA, neuronal development, mental retardation

## Abstract

Intellectual disability (ID) is one of the many features manifested in various genetic syndromes leading to deficits in cognitive function among affected individuals. ID is a feature affected by polygenes and multiple environmental factors. It leads to a broad spectrum of affected clinical and behavioral characteristics among patients. Until now, the causative mechanism of ID is unknown and the progression of the condition is poorly understood. Advancement in technology and research had identified various genetic abnormalities and defects as the potential cause of ID. However, the link between these abnormalities with ID is remained inconclusive and the roles of many newly discovered genetic components such as non-coding RNAs have not been thoroughly investigated. In this review, we aim to consolidate and assimilate the latest development and findings on a class of small non-coding RNAs known as microRNAs (miRNAs) involvement in ID development and progression with special focus on Down syndrome (DS) and X-linked ID (XLID) [including Fragile X syndrome (FXS)].

## Introduction

Intellectual disability (ID), also known as mental retardation, is the most common developmental disability and affects about 1–3% of the world population. It is a condition defined as having a significantly impaired cognitive ability and adaptive behaviors before the age of 18 (Daily et al., 2000). In clinical practice, diagnosis of ID in a patient is based on the intelligence quotient (IQ) below 70 at the age of 5 or older and deficits in two or more adaptive behaviors, such as communication, self-care, social skills, community access skills, self-direction, health, and safety (Kaufmann et al., 2007; Van Bokhoven, 2011). Conventionally, ID is divided into two distinctive groups; syndromic ID and non-syndromic ID. In syndromic ID, patients are presented with additional clinical features such as physical deformities or metabolic defects whereas intellectual impairment is the sole clinical features for non-syndromic ID (Kaufman et al., 2010; Van Bokhoven, 2011). However, there are considerable challenges in distinguishing both groups as some clinical features are very subtle and difficult to be diagnosed. Therefore, multiple comparisons have to be made between patients with common etiology to identify the genetic defects associated with the clinical features presented.

In addition to the classification based on clinical features, ID can be categorized according to the level of severity based on IQ of the patient. Patients with IQ below 20 is categorized as profound ID, IQ of 20–34 as severe ID, IQ of 35–49 as moderate ID, and IQ of 50–70 as mild ID (American Psychiatric Association, 2000). However, most studies often use a simpler classification where the patients are categorized as mild ID when the IQ lies between 50 and 70 or severe ID when the IQ is below 50. Under this simpler classification, only 20% of mild ID cases have any conclusive genetic causes whereas other cases are largely attributed by environmental factors such as malnutrition during pregnancy, infections, fetal alcohol syndrome, premature birth or exposure to neurotoxic compounds (Van Bokhoven, 2011). By contrast, about 50–65% of severe ID cases are commonly associated with genetic disorders resulted from chromosomal abnormalities, dysregulation of genetic imprinting, or monogenic disorders (Van Bokhoven, 2011).

The most prevalent genetic abnormalities associated with ID is chromosomal aberrations, such as trisomy 21 in Down syndrome (DS), trisomy 18 in Edward syndrome, the presence of extra chromosome X in males with Klinefelter syndrome or monosomy X in females with Turner syndrome, and chromosomal deletions at 22q11 in DiGeorge syndrome. Angelman syndrome and Prader–Willi syndrome are examples of genetic disorders caused by dysregulation of genetic imprinting, with ID as one of the common clinical features. On the other hand, monogenic disorder for ID is rare and commonly manifested as non-syndromic ID. It is also commonly known as X-linked ID (XLID) because most of the associated genes are located on the X chromosome. Coffin–Lowry syndrome is an example of XLID that arises as a result of X-linked dominant inheritance of mutated RPS6KA3 (ribosomal protein S6 kinase, 90 kDa polypeptide 3) on the X chromosome (Kleefstra et al., 2005).

Understanding the affected molecular pathways and cellular processes due to genetic abnormalities will help to unravel mechanisms underlying ID. However, the association between the genetic abnormalities and ID has remained unclear. In the past two decades, the discoveries of microRNAs (miRNAs), a class of small non-coding RNA about 18–24 nucleotides (nt), had brought new perspective to the studies of ID (Bartel, 2004). The discovery of a small RNA transcript *lin-4* in 1993 through a genetic screening in the nematodes *Caenorhabditis elegans* and its roles in regulation of *lin-14* translation via antisense RNA–RNA interaction have brought forth the beginning of the era of miRNAs and non-coding RNAs (Lee et al., 1993; Wightman et al., 1993). The era of miRNAs truly took off after the second miRNA, *let-7*, was discovered in 2000 in *C. elegans*. *Let-7* functions similarly as *lin-4* and was found to be conserved throughout metazoans (Reinhart et al., 2000). This finding sparks off a large scale searches for other miRNAs and their roles in regulating cellular and molecular processes (Lagos-Quintana et al., 2001; Lau et al., 2001; Lee and Ambros, 2001).

**Figure 1:**
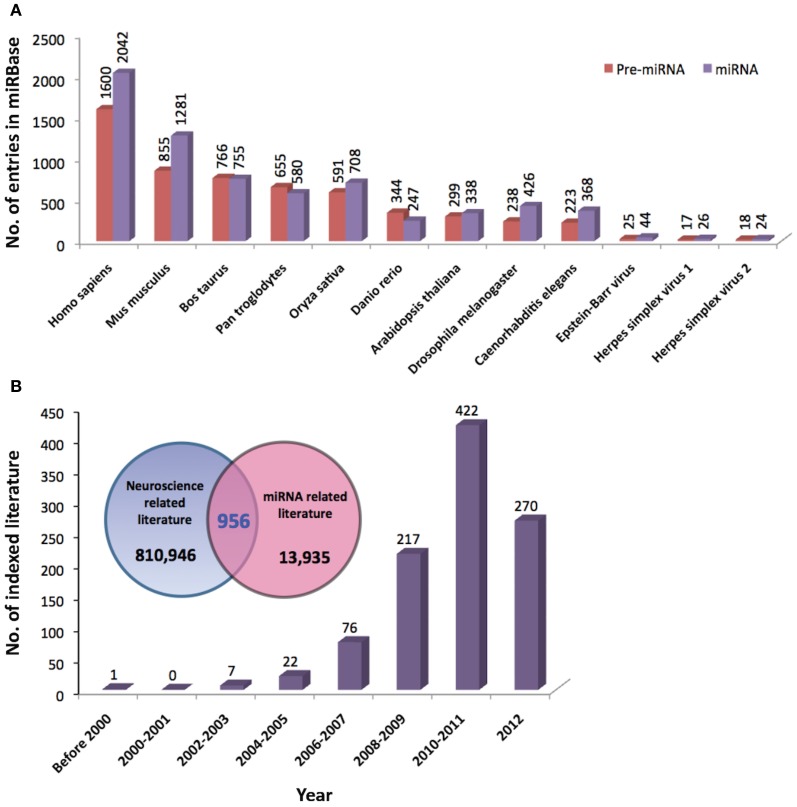
**(A)** Total number of precursor and mature miRNAs from various species of organisms indexed by miRBase (www.miRBase.org; miRBase v19 accessed on 8/11/2012). **(B)** The Venn diagram shows the total number of neuroscience- and miRNA-related literature indexed in PubMed. Neuroscience-related literature were searched based on the following terms/functions used in the title/abstract of the literature; (1) neuroscience or (2) brain or (3) neuron or (4) neuron or (5) glia or (6) nervous system. MiRNA-related literature were searched based on (1) miRNA or (2) microRNA or (3) *lin-4* or (4) *let-7* terms/functions. Total numbers of overlapping literature between the two groups of literature were searched based on the combined terms/functions: neuroscience-related search terms AND miRNA-related search terms. The graph shows the number of neuroscience-miRNA-related literature indexed in PubMed since 2000.

**Figure 2:**
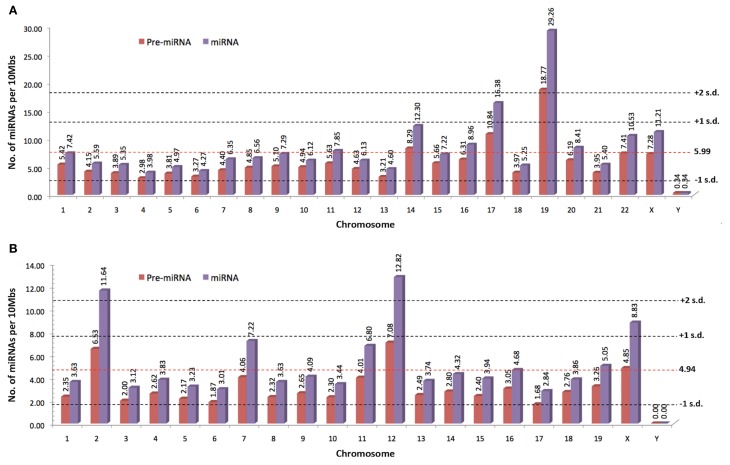
**The normalized number of precursor and mature miRNAs per 10 Mbs indexed by miRBase for (A) human and (B) mouse (www.miRBase.org; miRBase v19 accessed on 8/11/2012).** Red-dotted horizontal lines denote the average mature miRNAs per 10Mbs across the genome. Gray-dotted horizontal lines denote the number of standard deviation (SD) from the global average value.

Since the discovery of *lin-4* and *let-7*, a total of 21,264 miRNA entries were indexed in miRBase (www.mirbase.org; miRBase v19 accessed on 8/11/2012) for various organisms ranging from mammals (e.g., human, chimpanzee, mouse, cattle) to zebrafish, plants, arthropods, and viruses. Figure 1A shows the number of miRNAs indexed in miRBase v19 for selected organisms and the human genome harbors the greatest number of miRNAs that are reported to date (approximately 10% of all known miRNAs). In addition, the miRNA genes are not randomly distributed across the mammalian genome because some chromosomes in human (e.g., HSA19) and mouse (e.g., MMU2 and MMU12) genomes have exceptionally high density of miRNA genes as compared to the global average (Figure 2). The non-random genomic distribution of miRNA genes across the human genome or within a chromosome has been associated with dysregulated expression levels of miRNAs that may lead to diseases (Calin et al., 2004; Albano et al., 2010). Besides, changes of the copy number for miRNA genes due to aneuploidy may serve as one of the many causes of ID. Somatic mosaicisms in neurons due to aneuploidy have been identified in the mammalian brain and constitute only a small proportion of all neurons. These aneuploid neurons are resulted from errors in chromosomal segregation during normal progenitor cells proliferation and are integrated within circuitries of the normal brain (Rehen et al., 2001, 2005; Yang et al., 2003; Kingsbury et al., 2005). Taken together, the effect of non-random genomic distribution and imbalance dosage of miRNA genes in ID development may serve as an important piece of missing puzzle in understanding various genetic syndromes of ID.

To date, a great number of miRNAs have been shown to be associated with the development and function of nervous system (Christensen and Schratt, 2009; Fiore et al., 2011; Im and Kenny, 2012; McNeill and Van Vactor, 2012). The role of miRNAs in molecular regulation of gene expression in neurons (Siegel et al., 2011), learning and memory (Konopka et al., 2011), and the development of neurological disorders (Forero et al., 2010; Satoh, 2010) has been extensively reviewed. Since 2000, the total number of indexed literature in PubMed that are related to the field of neuroscience and miRNA has increased tremendously (Figure 1B). However, the role of miRNAs in ID has not gained much attention to date, thus in this review, we will provide a brief overview on miRNA biogenesis and function and highlight the involvement of miRNAs in molecular mechanisms related to ID with special focus on DS, XLID, and Fragile X syndrome (FXS).

## MiRNA biogenesis and function

**Figure 3:**
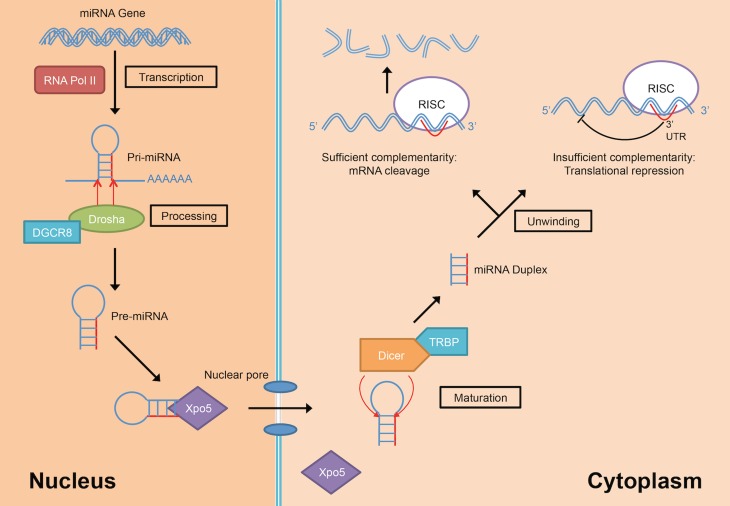
**MiRNA biogenesis and mode of action (DGCR8, DiGeorge Critical Region 8; RISC, RNA-induced silencing complex; RNA Pol II, RNA polymerase II; TRBP, Tar-RNA binding protein; Xpo5, Exportin-5)**.

MiRNAs are transcribed from their genes by either RNA polymerase II or III into a long primary transcript (pri-miRNAs) that folded itself into a hairpin structure (Figure 3) (Lee et al., 2004; Borchert et al., 2006). These miRNAs genes are located in both coding and non-coding genes across different regions in the genome. Frequently, miRNAs are transcribed from the introns of protein coding genes (Rodriguez et al., 2004; Saini et al., 2007). In addition, miRNAs are also transcribed from the non-coding region as part of long non-coding RNAs and are often arranged in clusters in the genome, leading to the formation of several miRNAs from the same pri-miRNA transcript (Rodriguez et al., 2004).

Following the transcription into pri-miRNA, the primary transcripts are then further processed in two stages by two different enzymes from the RNase III family; Drosha in the nucleus and Dicer in the cytoplasm. Drosha, in concert with DiGeorge Critical Region 8 (DGCR8) protein, facilitate the nuclear cleavage of pri-miRNAs into a ~60–90 nt stem loop intermediate resembles hairpin and termed as precursor miRNAs (pre-miRNAs) (Lee et al., 2003). The cleavage happened at the 5′ and 3′ arms of the pri-miRNAs hairpin by the action of two RNase domains on Drosha (Han et al., 2004). During the cleavage, DGCR8 is needed to stabilize the Drosha at its middle domain as well as to determine the exact cleavage site in the pri-miRNA. Subsequently, the resulted pre-miRNA hairpin is exported into the cytoplasm with the aid from a nuclear transport receptor, Exportin-5 (Xpo5) (Yi et al., 2003; Lund et al., 2004).

In the cytoplasm, pre-miRNAs are further processed into RNA duplexes of miRNA:miRNA^*^ by Dicer with the help of its cofactor, Tar-RNA binding protein (TRBP) (Chendrimada et al., 2005; Haase et al., 2005). There is an additional cleavage step for miRNAs that have high degree of complementarity along the hairpin stem by the action of Argonaute-2 (Ago2) which generates a nick in the middle of the prospective passenger strand (complementary strand to the mature miRNA that usually exists in low level), resulting in Ago2-cleaved pre-miRNA (ac-pre-miRNA) (Diederichs and Haber, 2007). The loop on both pre-miRNA and ac-pre-miRNA are then cleaved by Dicer to generate a miRNA duplex of about 22 nt in length.

The miRNA duplex generated from Dicer-mediated cleavage need to be separated into a functional guide strand that is complementary to the target and the passenger strand is usually degraded. The generation of the single stranded guide strand as the mature miRNA requires the unwinding activity of helicase. However, a universal helicase responsible for this has not been identified to date. The single stranded guide miRNA will be incorporated into a ribonucleoprotein effector complex, termed as RNA-induced silencing complex (RISC). Then, the miRNA directs the RISC to the target mRNA by binding to the 3′ UTR of the targeted mRNA. Nucleotides at positions 2–8 at 5′ region of the miRNA is known as the seed sequence and is involved in recognizing the target mRNA (Bartel, 2004). The choice of mRNA posttranscriptional regulation mechanisms depends on the complementarity of the seed sequence as well as supplementary binding of 3′ region of the miRNA with the 3′ UTR of the target mRNA. If the miRNA has perfect or sufficient complementarity to the mRNA, RISC will cleave off the target mRNA leading to mRNA degradation. Translational repression will happen instead if the miRNA:mRNA binding is imperfect (Bartel, 2004) leading to increased rate of degradation due to deadenylation of the mRNA (Eulalio et al., 2009). The deadenylation of mRNAs by miRNAs initiated the hypothesis that complete deadenylation leads to disruption of the interaction of poly(A)-binding protein and the cap, thus reducing the translation of the mRNAs. However, a recent publication demonstrated that in the case of *miR-430*, the translational repression occurred independent of the deadenylation (Bazzini et al., 2012). The author suggested that the miRNAs with partial complementarity to its target may trigger both translational repression and deadenylation to ensure maximum target mRNA repression and degradation.

## MiRNA and down syndrome

DS is a genetic disorder of trisomy human chromosome 21 (HSA21) with live birth prevalence of 11.2 per 10,000 births (Loane et al., 2012). All DS patients exhibit ID. They also develop other clinical features such as typical craniofacial appearance (e.g., brachycephaly, epicanthic fold and protruding tongue), hypotonia, congenital heart defects, early onset of Alzheimer's disease (AD), dementia as well as cognitive impairment (Van Cleve and Cohen, 2006; Van Cleve et al., 2006). Mild to severe intellectual disabilities were observed in DS patients with reported average value of 50 in IQ and learning disabilities involving both long-term and short-term memory formation (Brown et al., 2003; Vicari et al., 2005). The extra HSA21 caused various neuroanatomical abnormalities in DS patients such as reduction in brain size, brain weight as well as neuronal density, neuronal distribution, and dendritic abnormalities at cellular level (Wisniewski, 1990; Takashima et al., 1994; Kaufmann and Moser, 2000). There have been many hypotheses proposed to correlate the extra HSA21 to DS phenotypes such as gene dosage imbalance hypothesis, amplified developmental instability hypothesis, and critical region hypothesis (Delabar et al., 1993; Pritchard and Kola, 1999; Antonarakis et al., 2004). There is growing interest in epigenetic modifications, allelic differences, gene dosage compensation and also additive, subtractive, and epistatic patterns of genetic contribution to DS phenotypes (Dey, 2011).

Latest statistic from miRBase (www.mirbase.org; miRBase v19 accessed on 21/12/2012) indicates that there are 19 precursors and 26 mature miRNAs located on HSA21 but only five of them have been implicated in DS so far such as *miR-155, miR-802, miR-125b-2, let-7c*, and *miR-99a* (as summarized in Table 1) (Kuhn et al., 2008). These miRNAs are overexpressed in DS fetal hippocampus and heart samples and are exclusively expressed in neurons but not in microglial cells or astrocytes. Interestingly, neurons expressing these miRNAs were found increased by 10–15 fold in DS fetal hippocampus samples as compared to age- and sex-matched controls (Kuhn et al., 2008).

**Table 1:** **Summary of miRNAs that are associated with intellectual disabilities**.

**MicroRNA**	**Model/organism**	**Description**
**Target gene/signaling pathway**		
*miR-155*	DS individuals	Overexpressed in fetal hippocampus and heart samples (Kuhn et al., 2008).	
*MeCP2* (Keck-Wherley et al., 2011)	
*CFH* (Li et al., 2012)		Overexpressed in prefrontal cortex samples at all ages studied (from fetus to adulthood) (Kuhn et al., 2010).
*PICALM* (Paschou et al., 2012)		Overexpressed in the adult temporal lobe, the spleen, and the liver (Li et al., 2012).
	Ts65Dn mouse model for DS	Overexpressed in the adult hippocampus (Kuhn et al., 2010).
		Overexpressed in adult hippocampus and blood samples (Keck-Wherley et al., 2011).
		Anti-miR treatment restored MeCP2 in adult hippocampus (Kuhn et al., 2010).
	iPSCs generated from human DS amniotic fluid cells	Overexpressed in iPSCs (Lu et al., 2012).
	TNFα-stressed human neuronal-glial cells	Anti-miR treatment restored CFH level (Li et al., 2012).
	Superior temporal neocortex of AD	Overexpressed in neocortical samples (Lukiw and Alexandrov, 2012).
*miR-802*	DS individuals	Overexpressed in fetal hippocampus and heart samples (Kuhn et al., 2008).
*MeCP2* (Keck-Wherley et al., 2011)		Overexpressed in prefrontal cortex samples at all ages studied (from fetus to adulthood) (Kuhn et al., 2010).
	Ts65Dn mouse model for DS	Overexpressed in the adult hippocampus (Kuhn et al., 2010).
	Overexpressed in adult hippocampus and blood samples (Keck-Wherley et al., 2011).
		Anti-miR treatment restored MeCP2 in adult hippocampus (Kuhn et al., 2010).
	iPSCs generated from human DS amniotic fluid cells	Overexpressed in iPSCs (Lu et al., 2012).
*miR-199b*	Heart failure patients	Overexpressed in biopsies of cardiac tissues (Da Costa Martins et al., 2010).
*Dyrk1a* (Da Costa Martins et al., 2010)		
*SIRT1* (Saunders et al., 2010)		
	MHC-CnA mouse	Overexpressed in heart samples (Da Costa Martins et al., 2010).
		Anti-miR treatment restored Dyrk1a level in the heart (Da Costa Martins et al., 2010).
	Mouse embryonic stem cells	Overexpressed *miR-199b* reduced SIRT1 protein level (Saunders et al., 2010).
*miR-125b*	IL-6-stressed NHA	Overexpressed in IL-6-stressed NHA (Pogue et al., 2010).
*CDKN2A* (Pogue et al., 2010)		Anti-miR treatment restored CDKN2A level (Pogue et al., 2010).
*NR2A* and *EphA4* (Edbauer et al., 2010)		
*Nestin* (Cui et al., 2012)		
	Hippocampus of newborn	Overexpressed *miR-125b* reduced *Nestin* expression and inhibit neural stem/progenitor cells proliferation (Cui et al., 2012).	
	Sprague-Dawley rat pups
	E19 rat hippocampal neurons in culture	FMRP was partially depending on *miR-125b* in suppressing *NR2A* (Edbauer et al., 2010).
		Overexpressed *miR-125b* led to longer and thinner dendritic protrusions with reduced synaptic strength (Edbauer et al., 2010).
*miR-125b-2*	DS individuals	Overexpressed in fetal hippocampus and heart samples (Kuhn et al., 2008).
		Overexpressed in prefrontal cortex samples at all ages studied (from fetus to adulthood) (Kuhn et al., 2010).
*miR-99a*	DS individuals	Overexpressed in fetal hippocampus and heart samples (Kuhn et al., 2008).	
TGF-β pathway (Turcatel et al., 2012)	
*let-7c*		Overexpressed in prefrontal cortex samples at all ages studied (from fetus to adulthood) (Kuhn et al., 2010).	
TLP7 (Lehmann et al., 2012)	
	iPSCs generated from human DS amniotic fluid cells	Overexpressed in iPSCs (Lu et al., 2012).
*miR-214*	Ts65Dn mouse model for DS	Overexpressed in adult hippocampus samples (Keck-Wherley et al., 2011).
*CTTNBP2* and *ANK2* (Paschou et al., 2012)		
*Ncx1* (Aurora et al., 2012)		
	*miR-214* KO mouse	Increased NCX1 protein in heart samples (Aurora et al., 2012).
*miR-223*	Ts65Dn mouse model for DS	Overexpressed in adult hippocampus and blood samples (Keck-Wherley et al., 2011).	
*GluR2* and *NR2B* (Harraz et al., 2012)	
	XLID individuals	Mutation found in pre-miRNA sequence (Chen et al., 2007).
	P15–P20 *miR-223* KO mouse hippocampal neurons	Increased GluR2 and NR2B protein levels, and promoted NMDA-induced calcium influx (Harraz et al., 2012).
	P15–P20 *miR-223* WT mouse hippocampal neurons	Overexpressed *miR-223* caused NMDA-induced calcium influx inhibition (Harraz et al., 2012).
*miR-222*	XLID individuals	Mutation found in pre-miRNA sequence (Chen et al., 2007).
	Small RNA libraries from 26 different organ systems and cell types of human and rodents (Landgraf et al., 2007)	X-chromosomal miRNAs expressed in the midbrain, the hippocampus, and the cortex (Mendoza et al., 2010).
*miR-363*	XLID individuals	Mutation found in pre-miRNA sequence (Chen et al., 2007).
*FXR1P* (Cheever et al., 2010)		
	Small RNA libraries from 26 different organ systems and cell types of human and rodents (Landgraf et al., 2007)	X-chromosomal miRNAs expressed in the midbrain (Mendoza et al., 2010).
	HEK-293T cells	Downregulated the expression of *FXR1P* (Cheever et al., 2010).
*miR-221*	Small RNA libraries from 26 different organ systems and cell types of human and rodents (Landgraf et al., 2007)	X-chromosomal miRNAs expressed in the hippocampus and the cortex (Mendoza et al., 2010).
*let-7f-2*		X-chromosomal miRNAs expressed in the midbrain, the hippocampus, and the cortex (Mendoza et al., 2010).
*miR-19b-2*		X-chromosomal miRNAs expressed in the midbrain (Mendoza et al., 2010).
*miR-92a-2, miR-374b*		X-chromosomal miRNAs expressed in the midbrain and the hippocampus (Mendoza et al., 2010).
*miR-98, miR-374a*		X-chromosomal miRNAs expressed in the midbrain and the cortex (Mendoza et al., 2010).
*miR-105-2*		X-chromosomal miRNAs expressed in the cortex (Mendoza et al., 2010).
*miR-132*	E19 rat hippocampal neurons in culture	Overexpressed *miR-132* led to stubby and mushroom dendritic spines with an increase in average protrusion width as well as the synaptic strength (Edbauer et al., 2010).
*miR-92b, miR-367*	HEK-293T cells	Downregulated the expression of *FXR1P* (Cheever et al., 2010).
*FXR1P* (Cheever et al., 2010)		
*miR-9, miR-124*	*fxr1* KO mice	Both *pre-miR-9* and *pre-miR-124* required FXR1P to efficiently process them into mature miRNAs *in vitro* (Xu et al., 2011).
miR-19b, miR-302b^*^, miR-323-3p	HEK-293 cells	Potential repressors of the *FMR1* gene (Yi et al., 2010).
*FMR1* (Yi et al., 2010)		

15-LOX, 15-lipoxygenase; AD, Alzheimer's disease; ANK2, ankyrin 2; CDKN2A, cyclin-dependent kinase inhibitor 2A; CTTNBP2, cortactin-binding protein 2; CFH, complement factor H; DS, Down syndrome; Dyrk1a, dual specificity tyrosine-phosphorylation-regulated kinase 1A; E19, embryonic day 19; EPHA4, Ephrin type A receptor 4; FMR1, fragile X mental retardation 1; fxr1, Farnesoid X receptor 1; FXR1P, fragile X related protein 1; GluR2, glutamate receptor, ionotropic, AMPA2 (alpha 2); IL-6, interleukin-6; iPSC, induced pluripotent stem cell; KO, knockout; MeCP2, methyl CpG binding protein 2; MHC-CnA, myosin heavy chain-calcineurin; Ncx1, sodium-calcium exchanger 1; NHA, normal human astrocytes; NMDA, N-methyl D-aspartate; NR2A, NMDA subunit 2A; NR2B, NMDA subunit 2B; P, postnatal; PICALM, phosphatidylinositol-binding clathrin assembly protein; SIRT1, sirtuin1; TGF-β, transforming growth factor-β; TLP7, toll-like receptor 7; TNF, tumor necrosis factor; UTR, untranslated region; WT, wildtype; XLID, X-linked intellectual disability;

* *denotes the passenger strand of a miRNA*.

Trisomic overexpression of HSA21-derived miRNA is believed to contribute, in part, to the defective neurodevelopment in DS individuals. Overexpression of *miR-155* and *miR-802* (Table 1) in Ts65Dn (a genetic mouse model of DS) hippocampus may contribute to impaired hippocampal synaptic plasticity and neurogenesis (Keck-Wherley et al., 2011). Both *miR-155* and *miR-802* target the methyl CpG binding protein 2 (*MeCP2*) (Kuhn et al., 2010), which is associated with deleterious effects on development as seen in Rett Syndrome (Cohen et al., 2003). Silencing of these two miRNAs in Ts65Dn is able to restore the *MeCP2* expression and its target genes (Kuhn et al., 2010). Overexpression of *miR-155* and *miR-802* and low expression of *MeCP2* were observed in induced pluripotent stem cells (iPSC) from human DS amniotic fluids cells (Lu et al., 2012). Interestingly, a study in mouse model of Rett Syndrome showed that *MeCP2* repressed *miR-199b* expression in the mouse brain (Urdinguio et al., 2010) and *miR-199b* has been shown to suppress sirtuin 1 (SIRT1) (Saunders et al., 2010), which is involved in dendritic development (Codocedo et al., 2012). Reduction of SIRT1 promotes neurogenic potential of adult subventricular zone and hippocampal neural precursors (Saharan et al., 2013). As aforementioned, overexpression of *miR-155* and *miR-802* have been found to repress *MeCP2* expression (Kuhn et al., 2010), therefore these HSA21-miRNAs in DS brain are expected to repress *MeCP2* and subsequently relieve the suppression on *miR-199b* and decreased SIRT1 expression. This interconnected cascade of regulations suggests a series of complex events that may be disrupted in DS brain development. Selective inactivation on these HSA21-derived miRNAs can be employed as a novel therapeutic tool in the future.

DS is also characterized by brain deficits and systemic immune pathology such as activated microglia and increased inflammatory signaling. Overexpression of *miR-155* and repression of complement factor H (CFH), an essential repressor of the innate immune response were observed in brains and peripheral tissues of DS individuals (Li et al., 2012). Interestingly, *miR-155* was overexpressed in AD and was found to regulate CFH expression (Lukiw and Alexandrov, 2012). It was predicted to interact with presynaptic mRNAs such as phosphatidylinositol-binding clathrin assembly protein (*PICALM)* (Paschou et al., 2012), which has been implicated in AD (Melville et al., 2012). As DS patients are prone to develop early onset AD, association between *miR-155* and brain pathology should be further investigated to unravel its role in regulating innate immune and inflammatory responses.

There have been efforts made to unravel the role of *miR-125b* (summarized in Table 1) in the neuropathology of DS subjects. *MiR-125b* was significantly upregulated in interleukin-6-stressed normal human astrocytes (Pogue et al., 2010). Anti-*miR-125b* treatment attenuated astrogliosis and increases cyclin-dependent kinase inhibitor 2A (CDKN2A), a negative regulator of cell growth. Astrogliosis in DS brains could be attributed to the glial proliferative effect of *miR-125b* via suppression of CDKN2A (Pogue et al., 2010). In addition, *miR-125b* also targeted glutamate receptor, ionotropic, *N*-methyl d-aspartate subunit 2A (*NR2A)*, Ephrin type A receptor 4 *(EPHA4)*, and *Nestin* (Edbauer et al., 2010; Cui et al., 2012). *Mir-125b* is crucial for spine morphology development and neuronal survival (Schaefer et al., 2007). It stimulates neurite outgrowth and dendritic branching (Le et al., 2009) which produced longer and thinner dendritic spine with reduced spine width by targeting *NR2A* (Edbauer et al., 2010). Suppression of NR2A and EphA4 have been implicated in long-term potentiation (LTP) and/or long-term depression (LTD) processes (Zhao and Constantine-Paton, 2007; Filosa et al., 2009; Xu et al., 2009). In a Luciferase reporter assay, *miR-125b* was shown to target 3′UTR of *Nestin* and reduced both *Nestin* mRNA and protein levels. When overexpressed, *miR-125b* inhibited neural stem/progenitor cells proliferation via suppression of *Nestin* (Cui et al., 2012). Taken together, it is evident that the overexpression of *miR-125b* may lead to significant changes in neuronal development. The role of *miR-125b* in targeting various transcripts leading to impaired synaptogenesis and LTP in DS subjects should be investigated.

*Mir-99a* regulates transforming growth factor-β (TGF-β) pathway (Turcatel et al., 2012) which directly involved in retrograde synaptic signaling (Sanyal et al., 2004) and regulates Wnt signaling in stem cell differentiation (Cai et al., 2013). On the other hand, *let-7c* is highly expressed in the hippocampal region of adult mice (Bak et al., 2008). It has been shown to cause neuronal loss in a dose- and time-dependent manner by acting on toll-like receptor 7 (TLR7) (Lehmann et al., 2012). Role of *mir-99a* and *let-7c* in regulating neuronal function in DS individuals warrant further investigations.

Children with DS often experience auditory deficits (Cunningham and McArthur, 2006), language difficulties which include reading impairment (Lemons and Fuchs, 2010; Nash and Heath, 2011) and limited verbal short-term memory capacity (Jarrold et al., 2002; Brock and Jarrold, 2005). These DS-associated impairments will generally lead to learning disabilities in DS children. *MiR-125b-2*, *let-7c*, and *miR-99a* are the three HSA21-derived miRNAs which orthologs were found expressed in the mouse inner ear (Weston et al., 2006). An increased in the size of the large conductance calcium activated potassium (BK) current (which is known to determine electrical tuning) was observed in Tc1 (another mouse model for DS that carries a significant portion of HSA21) inner hair cells as compared to control (Kuhn et al., 2012). Thus, the roles of *miR-125b-2*, *let-7c*, and *miR-99a* should be validated in human samples to better understand their roles in regulating auditory development or function in DS individuals.

Other non HSA21-derived miRNAs such as *miR-214* and *miR-223* were found overexpressed in the adult Ts65Dn hippocampus (Keck-Wherley et al., 2011) but their expression profiles have not been extensively characterized in DS individuals. *Mir-214* was predicted to regulate cortactin-binding protein 2 *(CTTNBP2)* and ankyrin 2 *(ANK2)* that code for synaptic protein (Paschou et al., 2012). It also repressed sodium-calcium exchanger 1 (*Ncx1)* (Aurora et al., 2012), which is crucial for spine-dendrites compartmentalization (Lörincz et al., 2007) and presynaptic calcium recovery mechanism in cerebellar parallel fiber (Roome et al., 2013). *MiR-223* has been reported to function as a regulator on glutamate receptor subunits NR2B and GluR2 and its overexpression led to NMDA-induced calcium influx inhibition on hippocampal neurons (Harraz et al., 2012). Downstream gene targets of both *miR-214* and *miR-223* have important roles in regulating neuronal development and function, however, the direct association between these miRNAs and their targets with neuronal dysfunction in DS are yet to be determined.

MiRNAs derived from other chromosomes but targeted genes located on HSA21 may contribute to the phenotypes of DS. For instance, both mouse chromosome 9-derived *miR-199b* and human chromosome 2-derived *miR-1246* are known to downregulate DS associated dual-specificity tyrosine-(Y)-phosphorylation regulated kinase 1A gene (*Dyrk1a*—encoded in MMU16; *DYRK1A*—encoded in HSA21) and subsequently induce apoptosis through nuclear factor of activated T-cells (NFAT) pathway (Da Costa Martins et al., 2010). *Dyrk1a* with dysregulated NFAT signaling has been reported in DS individuals (Arron et al., 2006). NFAT signaling pathway has been implicated in AD (Abdul et al., 2009; Hudry et al., 2012). Based on the AD model, further investigation of both *miR-199b:Dyrk1a* and *miR-1246:DYRK1A* interactions may provide an insight on mechanisms mediated by these miRNAs in ID progression among DS individuals.

In brief, HSA21-harbored miRNAs are directly involved in neuronal differentiation, neuronal development, synaptic plasticity and more. Their roles in DS may shed light in understanding the underlying mechanisms in the development of ID More excitingly, miRNA-based therapies have been shown to restore expression of targeted genes as well as their downstream activities. Restoration of defective neurogenesis, gliogenesis and cell cycles events have not been fully explored and these warrant further investigation to better understand ID development in DS patients.

## MiRNA and X-linked intellectual disabilities

X chromosome plays a major role in the development of sexual characteristics and it harbors many genes that are involved in cognitive functions. XLID broadly refer to different forms of ID that are inherited as X-linked traits. First reported in 1938, Lionel Penrose observed higher number of males suffered from ID than female from a survey and classification of those in institutional care and their relatives (Penrose, 1938). He also found that the ratio between male to female ID patients was 1.25:1, which was eventually confirmed by many other studies in USA, Canada, Australia as well as Europe with all the studies came into agreement that about 30% excess of males as compared to female were affected by ID (Raymond, 2006). From various family studies, it was then discovered that many of the ID was inherited as X-linked trait. Hence, XLID typically affects the male population with mild to severe ID. Females are less likely to be affected by XLID. If they are affected, they developed milder ID symptoms than the male. This is due to X chromosome inactivation where one of the two X chromosomes in female is inactivated randomly early in development. The X-inactivation is a dosage correction mechanism in female to prevent the double dosage effect of the genes on both X chromosomes. For female with XLID, the mechanism could reduce the effect of mutated or deleted genes by inactivating the affected X chromosome.

XLID is manifested in more than 150 syndromes, including FXS, Klinefelter syndrome, Rett syndrome, Coffin–Lowry syndrome, and X-linked alpha thalassemia. To date, 102 X-linked genes have been associated to 82 syndromes with mutations in these genes described as the causes (Stevenson et al., 2012). However, the identification of genes that are responsible for other XLID syndromes had been unsuccessful due to the rarity of the syndromes or the difficulty in identifying the genes involved. This has impeded the progression in XLID related research.

In 2007, a large-scale mutation screening of miRNAs in patients with ID was reported (Chen et al., 2007). A cohort of 464 patients with XLID has been screened for mutations in 13 known, brain expressed X-chromosomal miRNAs. Four nucleotide changes in three different pre-miRNAs (*miR-222*, *miR-223*, and *miR-363*; Table 1) have been observed in the study though the changes appeared to be functionally neutral and did not affect the function of the respective miRNAs. The authors suggested that the functionally neutral mutations on the miRNAs indicate a strong selection on the pre-miRNA and thus reflecting on the general importance of miRNA system.

Mendoza and colleagues are interested in the potential roles of brain expressed X-chromosomal miRNAs especially in the context of human intelligence and they attempted such investigation by using informatics tools (Mendoza et al., 2010). By mining the Mammalian miRNA Expression Atlas based on Small RNA Library Sequencing (Landgraf et al., 2007), 77 human X-chromosomal miRNAs was identified and ten were further selected for target genes prediction as well as gene ontologies (GO) analysis by virtue of their differential levels in the cortex, hippocampus and midbrain. The ten miRNAs selected were *let-7f-2*, *miR-19b-2*, *miR-92a-2*, *miR-98*, *miR-105-2*, *miR-221*, *miR-222*, *miR-363*, *miR-374a*, and *miR-374b* (Table 1). Out of the 10 miRNAs, only *let-7f-2* and *miR-222* were expressed in all the three brain regions studied indicating that they may play an important role in regulating brain function. Target genes for all the 10 selected miRNAs were predicted using *in silico* tools (MiRanda, TargetScan, and MirTarget2) and results from all analyses were pooled for GO annotation enrichment analysis. The enrichment analysis showed that many biological processes were associated with brain development including the development of the forebrain, midbrain as well as hippocampus. These results show that X chromosome is a potential repository for genes highly expressed in the brain and may have roles in regulating the development or function of nervous system.

## MiRNA and fragile X syndrome

FXS, also known as Martin Bell syndrome, is one of the most common forms of XLID with an estimated prevalence of 1 in 4000 males and 1 in 8000 females (Warren and Sherman, 2001). The syndrome is transmitted as an X-linked dominant trait and individuals with the syndrome are presented with mild to severe ID (IQs in the range of 20–70), mild abnormal facial features (mainly in males), as well as macroorchidism in postpubescent males (Warren and Nelson, 1994). The loss-of-function mutation on fragile mental retardation 1 (*FMR1*) gene, due to the trinucleotide expansion of CGG repeat in 5′ untranslated region (UTR), has been identified as the cause for FXS. The large trinucleotide expansion has been associated with hypermethylation of both the CGG repeat and upstream CpG islands, resulting in transcriptional silencing of *FMR1*, which leads to the loss of its product, fragile X mental retardation protein (FMRP) (Jin et al., 2004a). As a translation repressor, miRNAs may play a role in downregulating the production of FMRP. For example, *miR-19b*, miR-302b^*^, and *miR-323-3p* (Table 1) were shown to target 3′ UTR of *FMR1* in a luciferase reporter gene assay (Yi et al., 2010). This suggests that the expression of FMRP is susceptible to miRNA regulation by targeting *FMR1* transcripts. Such incident, however, have not been demonstrated or validated in FXS subjects to date.

FMRP is an RNA-binding protein which plays a role in mRNA transport and translational regulation especially in the synaptic region of neurons. Loss of functional FMRP impairs normal synaptic plasticity, which is believed to be the molecular basis for ID in FXS patients (Bassell and Warren, 2008). Comparing miRNAs targets predicted by miRanda with datasets of FMRP-bound mRNAs, 74% of the mRNAs were found to be matching with miRNAs target genes (John et al., 2004). This suggests that some of the translation may be regulated through actions of both FMRP and miRNAs, indicating a close association between both. Indeed, it was shown that FMRP interacts with miRNAs as well as its machinery including Dicer and mammalian ortholog of Argonaute 1 (AGO1) in mammalian cell cultures (Jin et al., 2004b). FMRP also facilitate the assembly of miRNAs on specific target mRNAs by acting as a miRNA acceptor protein for Dicer (Plante et al., 2006). RNAs targeted by FMRP were involved in synaptic signaling pathways such as synaptic LTP, glutamate receptor signaling, neuropathic pain signaling, GABA receptor signaling, synaptic LTD and CREB signaling in neurons. FMRP bound to its RNA targets mainly at the coding sequences and stalled ribosomes (Darnell et al., 2011). Loss of FMRP function relieved the ribosomal stalling, thus suggesting that FMRP may regulate translation at the level of elongation (Darnell et al., 2011). Since FMRP could act as a miRNA acceptor from Dicer, any defect in the function of FMRP will not only cause abnormalities in miRNA function but translational regulation of its targeted mRNAs as well.

FMRP-associated *miR-125b* and *miR-132* have a crucial role in regulating the structure and function of the synapses (Edbauer et al., 2010). By overexpressing *miR-125b* in mouse hippocampal neurons in culture, longer and thinner dendritic protrusions with reduced synaptic strength were observed. Overexpression of *miR-132*, however, resulted in stubby and mushroom spines with an increased average protrusion width as well as the synaptic strength (Table 1). When FMRP was downregulated *in vitro*, overexpression of both *miR-125b* and *miR-132* did not cause any phenotypic changes to the dendritic spine morphology suggesting both FMRP and its associated miRNAs were needed for translation regulation. Together with FMRP, *miR-125b* targeted and suppressed NR2A, a subunit of NMDA (*N*-methyl-D-aspartate) receptor (Edbauer et al., 2010), which has an important role in LTP in cortex and amygdala, spike-timing-dependent plasticity and ocular dominance plasticity (Li et al., 2002; Zhao et al., 2005; Desai et al., 2006; Dölen, 2007; Meredith et al., 2007). All these findings further solidified the potential role of *miR-125b* and FMRP in the synaptic plasticity and function.

In addition to FMRP, another paralogs of FMRP known as Fragile X-related protein 1 (FXR1P) was targeted and regulated by *miR-92b*, *miR-363*, and *miR-367* (Table 1). Overexpression of *miR-367* downregulated the expression of FXR1P in human HEK-293 and HeLa cell lines (Cheever et al., 2010). Interestingly, FXR1P was required to efficiently process *pre-miR-9* and *pre-miR-124* into mature miRNAs *in vitro*. Loss of FXR1P led to the downregulation of the brain specific miRNAs, that were crucial to several aspects of neuronal development and function (Xu et al., 2011). Mutations in either miRNA recognition sites for *miR-92b*, *miR-363*, or *miR-367* on FXR1P will result in increased levels of FXR1P that leads to a lower level of *miR-9* and *miR-124*. Collectively these findings imply that FXR1P are far more important than previously thought due to the facts that FXR1P functions in processing pre-miRNAs and yet FXR1P itself are regulated by miRNAs. This central role of FXR1P leads us to postulate that mutations on FXR1P may contribute to FXS essentially due to disrupted miRNA-mediated translation regulation.

To date, there are no conclusive evidences that showed direct link between miRNAs and FXS. However, the prospect of miRNAs in answering much of the question about FXS continues to encourage researchers in pursuing miRNA as a potential breakthrough to understand FXS. With its role as a regulator in the expression of multitude of genes, miRNAs definitely will help in filling much of the gaps in our understanding of XLID.

## Conclusion

As much as we have progressed in the discovery of genes or mechanisms responsible for ID, many other syndromes where ID was implicated remained elusive and under-investigated. These pose a great challenge to researchers in their effort to better understand the “universal” mechanism underlying the development and progression of broad-spectrum and heterogenous ID conditions. In present day, miRNAs have the potential to bridge the missing link between genetic abnormalities with ID especially in cases where the causative genes could not be identified. In addition, miRNAs' unique characteristics of being able to target multiple genes or that a single gene could be targeted by multiple miRNAs may explain the reason behind some severe ID conditions where multiple genes are affected. The one-to-one, one-to-many, and many-to-one mode of miRNA:mRNA interactions provide us with a great versatility in designing experiments to study the development and progression of ID as well as develop therapeutic strategies aiming at pharmacologic inhibition of ID-associated miRNAs. Besides drug based approach, various molecular therapies based on small interfering RNA (siRNA) and locked nucleic acid (LNA) have been tested and hold promises in treating genetic disorders (Frieden and Ørum, 2008; Fluiter et al., 2009; Bernardo et al., 2012; Chabot et al., 2012). In addition, the advent of nanotechnology-based delivery system as well as advancement of stem cell researches would make the goal of treating and improving the cognitive function of individuals with ID a reality in near future.

### Conflict of interest statement

The authors declare that the research was conducted in the absence of any commercial or financial relationships that could be construed as a potential conflict of interest.
